# Effects of Lemon Beverage Containing Citric Acid with Calcium Supplementation on Bone Metabolism and Mineral Density in Postmenopausal Women: Double-Blind 11-Month Intervention Study

**DOI:** 10.1155/2021/8824753

**Published:** 2021-02-24

**Authors:** Hiromi Ikeda, Tadayuki Iida, Masanori Hiramitsu, Takashi Inoue, Satomi Aoi, Miho Kanazashi, Fumiko Ishizaki, Toshihide Harada

**Affiliations:** ^1^Faculty of Health and Welfare, Prefectural University of Hiroshima, Mihara 723-0053, Japan; ^2^Research & Development Division, Pokka Sapporo Food & Beverage Ltd., Yaizu 425-0013, Japan; ^3^Prefectural University of Hiroshima, Mihara 723-0053, Japan

## Abstract

A critical factor for preventing osteoporosis after menopause is attenuation of the accelerated turnover rate of bone metabolism. The present randomized controlled study was conducted to clarify the effects of a lemon beverage with calcium (Ca) supplementation that makes use of the chelating action of citric acid. Comprehensive evaluations of bone were performed by assessments of bone mineral density (BMD) and biomarkers related to bone turnover. Seventy-nine postmenopausal women were enrolled and asked to participate in an 11-month continuous intake of the test beverages. The subjects were divided into three groups: those who consumed a lemon beverage containing citric acid with Ca supplementation (LECA group), those who consumed a lemon beverage containing citric acid without Ca supplementation (LE group), and those who consumed no test beverage (control group). Using a double-blind protocol, subjects in the LECA and LE groups consumed one bottle containing 290 mL of the test beverage each day. The ratio of change in BMD after 11 months was significantly higher in the LECA group as compared to the control and LE groups. The LECA group also showed significant decreases in concentrations of tartrate-resistant acid phosphatase 5b (TRACP-5b), a bone resorption marker, and bone alkaline phosphatase (BAP) as compared to the other groups, as well as a significant decrease in concentration of osteocalcin (OC), a bone formation marker, as compared to the LE group. Based on our findings, we speculated that bone resorption and bone formation in postmenopausal women might be suppressed along with an increase in Ca resorption caused by chelation of citric acid in association with continuous ingestion of a Ca-supplemented lemon beverage containing citric acid, resulting in suppression of high bone metabolic turnover. In addition, the results provide information regarding BMD maintenance in the bones of the trunk, including the lumbar spine and proximal femur.

## 1. Introduction

The incidence of osteoporosis is exceptionally high in postmenopausal women, as a low estrogen level associated with menopause can lead to insufficient suppression of bone resorption. The result is increased bone resorption and consequently decreased bone mass, resulting in postmenopausal osteoporosis [1], while related osteoporotic fractures are associated with a higher risk of mortality [2]. A critical factor in preventing osteoporosis after menopause is attenuation of the accelerated turnover rate of bone metabolism. A variety of studies regarding the prevention of osteoporosis have been reported. Among the factors examined, lifestyle-related behavior, especially dietary and fitness habits, is considered to be important [3–5]. Furthermore, the acid load imposed by a modern diet is thought to play an important role in the pathophysiology of osteoporosis. Recently, several studies have investigated the effects of nutrients on bone metabolism and bone mineral density (BMD), with the chelation of citric acid receiving attention in this field [6–8]. Those studies indicated that the chelating action of citric acid induces an increase in resorption of potassium and calcium (Ca), leading to the induction of beneficial effects toward BMD and metabolism. However, no known studies regarding the effects of Ca absorption of citric acid on BMD and bone metabolism in healthy subjects have been reported.

We previously conducted a 5-month interventional trial using lemon beverages containing approximately 1.5 g of citric acid in each bottle (290 mL), with that consumed by the experimental group supplemented with 350 mg Ca, which were consumed by postmenopausal women under a double-blind protocol [9]. Those findings indicated that citric acid in lemon possibly stimulates Ca resorption, resulting in suppression of increased bone metabolism and maintenance of BMD in the lumbar spine. However, no statistically significant differences were noted in the proximal femur in subjects in previous studies, which may have been because of differences in the ratio of cancellous bone between the lumbar spine and femur [10, 11]. For evaluating factors related to bone fracture caused by osteoporosis, BMD assessment of trunk bones, such as the lumbar spine and femur, using dual energy X-ray absorptiometry (DEXA) has been recommended [12, 13]. Furthermore, previous interventional studies of subjects administered therapeutic medicine have noted increases in BMD over a 1-year time frame [14, 15]; thus, evaluation after a greater period of consumption is required. As a result, a longer intervention period along with BMD evaluation of trunk bones by DEXA might be necessary for validation of these findings.

A systematic review of calcium intake and fracture risk by Bolland et al. [16] found no evidence of reduced risk of fractures in the hip and forearm. Furthermore, Rebecca et al. [17] reported that hip, vertebrae, forearm, and wrist fracture rates in subjects receiving supplementation with Ca carbonate (up to 1000 mg/day) and vitamin D (up to 600 IU/day) were not significant as compared to the placebo group. In addition, those reports noted increased risk of kidney stones in association with Ca intake. These findings are in contrast to the effects of Ca intake on trunk bone fractures; thus, a more comprehensive understanding of prefracture bone mineral density and the effects of Ca on bone metabolism is needed, while its impact on increased risk of kidney stones must be considered.

The present study was conducted to compare a standard intake of Ca with the recommended intake in Japanese subjects to verify the effects of consumption of a lemon beverage with and without sufficient Ca in order to closely match conditions similar to daily life. In order to verify bone mineral density of trunk bones and the effects on bone metabolism, a comprehensive evaluation of bone was performed using measurements of BMD and biomarkers related to bone turnover in the lumbar spine and proximal femur. The results thus obtained clarify the effects of long-term intervention with a Ca-supplemented lemon beverage on bone metabolism in healthy postmenopausal women.

## 2. Materials and Methods

### 2.1. Subjects

Healthy postmenopausal women were recruited as trial volunteers by placing a public notice in the newsletter of M City, Japan. The exclusion criteria were (1) individuals with serious comorbidities, such as myocardial or cerebral infarction, or hepatic or renal insufficiency; (2) those being administered warfarin, because of possible effects of the test beverages on drug efficacy; and (3) those judged to be unsuitable by the principal investigator. The participants received a detailed explanation regarding the contents and methods of the present study at an information meeting held prior to the beginning of the trial. Informed written consent was obtained from 100 volunteers.

The present study was conducted as a randomized control trial using methods previously reported by Hua et al. in 2018 [7, 9]. The subjects were given individual ID numbers to assure anonymity. After matching by age (±3 years) and body mass index (BMI) (±3 kg/m2), they were divided into the LECA group, which received a Ca-supplemented lemon beverage containing citric acid (*n* = 28), and the LE group, which received a lemon beverage containing citric acid without Ca supplementation (*n* = 28) (researchers: T. Iida, H.I.), as well as a control group, which received no lemon beverage (*n* = 23 subjects) (researcher: M.H.). The years after menopause in subjects in each group at the time of enrollment were noted, as follows. The researchers were blinded to which subjects were allocated to the groups as well as data analysis related to allocation. Control group subjects were not given a placebo and asked to conduct themselves normally throughout the study period. For the LECA and LE groups, the test beverages were distributed using a double-blind method.

This study was performed after receiving approval from the Ethics Committee of the Prefectural University of Hiroshima (No. 15MH025-01) and the protocol used was in accordance with the Declaration of Helsinki. The study has been registered in the UMIN Clinical Trials Registry (No. UMIN000018952).

## 3. Items Investigated

The period of the intervention was from December 2015 to December 2016. During that time, subjects in the LECA and LE groups were asked to consume one bottle of the test beverage (290 mL) each day continuously for 11 months. Physical BMD and bone metabolism-related factors were measured at the start of the study, just prior to starting the test beverages, and again after 5 and 11 months of consumption at the Mihara Campus of Prefectural University of Hiroshima.

### 3.1. Physical Measurements

Age and years after beginning menopause were determined by interview, while body height, weight, and BMI were obtained by physical measurements.

### 3.2. BMD

BMD (g/cm2) was determined in examinations of the lumbar spine (L2 to L4) and left proximal femur using an X-ray bone densitometer (Discovery, Toyo Medic Co., Ltd.) [12, 18]. Based on calibrations repeated 99 times using the same equipment during the intervention period, mean BMD was 1.038 g/cm2, with a standard deviation (SD) of 0.03 g/cm2 and cv of 0.37%, which fell within the normal ranges established by Hologic, Inc.

### 3.3. Bone Metabolism-Related Items

For bone resorption markers, serum tartrate-resistant acid phosphatase type 5b (TRACP-5b) and urinary type 1 collagen cross-linked N-telopeptide (u-NTx) levels were determined, while bone alkaline phosphatase (BAP) and osteocalcin (OC) were examined as bone formation markers and undercarboxylated osteocalcin (ucOC) as a bone matrix-related marker. As for other factors related to bone metabolism, highly sensitive parathyroid hormone (HS-PTH) and serum total Ca concentrations were determined. Blood samples for these tests were collected at least two hours after eating a meal.

Serum samples were obtained and prepared by centrifugation at 3000 rpm for 7 minutes; then, TRACP-5b, BAP, OC, ucOC, highly sensitive PTH, and serum total Ca were measured using commercially available kits. Urine samples for u-NTx determination were examined using a commercially available kit without pretreatment after collection. All serum and urine samples were analyzed at Fukuyama Medical Laboratory. TRACP-5 b in serum was determined using Osteolinks TRAP-5b (Nittobo Medical Co., Ltd.), BAP using Access Ostase (Beckman Coulter Co. Ltd.), OC using BGPIRMA LSI-M (LSI Medience Co., Ltd.), and ucOC using Picolumi ucOC (Eidia Co., Ltd.). For u-NTx determination, OSTEOMARK (Alere Medical Co., Ltd.) was used. Highly sensitive PTH was measured using a YAMASA PTH kit (Yamasa Corporation) and serum total Ca concentration with SEROTEC CaAL Type C (Serotec Co., Ltd.), an Arsenazo III-based assay.

### 3.4. Composition of Test Beverages and Consumption Method

The test beverages were provided by POKKA SAPPORO Food & Beverage Ltd., a collaborating research institution. The composition of each is shown in Table 1. The subjects were instructed to drink one bottle (290 mL) of the test beverage each day. Timing was not designated. In addition, they were asked to keep a consumption record sheet, which was collected every three months. The mean rate of daily consumption for all subjects was 94.2%. The test beverages for the LECA and LE groups contained 30 mL of lemon juice, while the Ca-supplemented lemon beverage given to the LECA group also contained 350 mg of Ca.

### 3.5. Data Analysis

**Figure 1:**
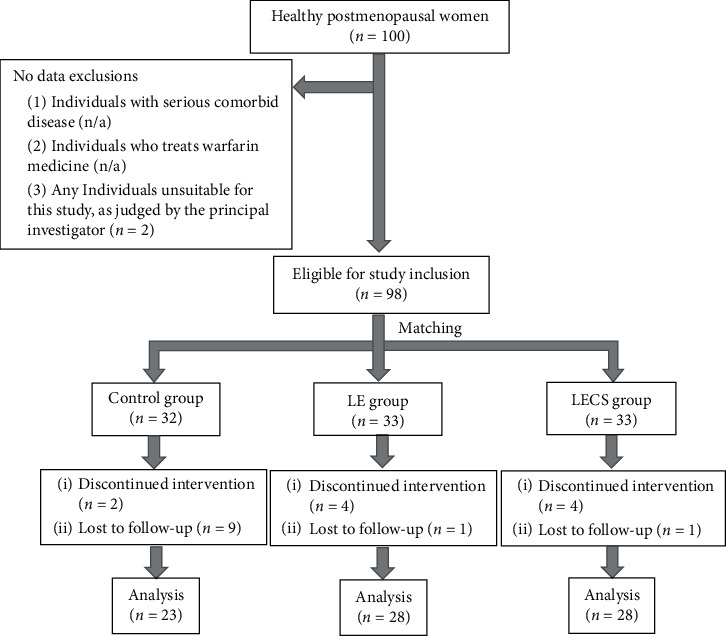
Study enrollment flowchart. The LE group received a nonsupplemented lemon beverage containing citric acid. The LECA group received a lemon beverage containing citric acid supplemented with Ca.

The sample size was determined according to the 6-month difference regarding bone mineral density in the lumbar spine using G-power. Based on the results of a prior study conducted by Ikeda et al. [9] on the relationship between an LECA group and control group and after considering *M*1 = 0.021 (mean BMD of 6 months, difference in the control group), SD 1 = 0.020, *M*2 = -0.027 (mean BMD of 6 months difference in the LECA group), SD2 = 0.041, two-sided *α* = 0.05, and power = 95%, the minimum number of subjects per group was determined to be 15, which was increased to 23 (minimum sample size of the control group) to take account for potential attrition, making the total sample size 79. Thus, 79 individuals who participated in all medical examinations conducted at the start of the trial prior to consuming a test beverage, as well as 5 and 11 months after starting consumption ingestion, were subjected to analysis. The subjects included those who had not been diagnosed with osteoporosis but were at a level indicating such a diagnosis. The study flowchart is shown in Figure 1. Obtained data were tested for normality using a Shapiro-Wilk test. Values for investigated items obtained before starting consumption of the test beverages are expressed as the mean (SD) when normality was confirmed and as the median (25%–75%) when normality was not confirmed. As for the relationship between the test beverage and period of consumption for the investigated items (BMD, bone metabolism marker levels), when normality was confirmed, a two-way analysis of variance was performed after adjusting for age and BMI in all three groups (LECA, LE, and control), as well as intervention period at the start of the trial just prior to the first consumption of the test beverage ingestion. The same analysis was performed again at 5 and 11 months after starting the trial, with BMI and bone metabolism marker levels used as dependent variables. When normality was not confirmed, the results of each group were subjected to Friedman's test. Furthermore, the ratio of change in BMD and bone metabolism marker levels was calculated by subtracting the initial value from that after 11 months and dividing by the initial value. When normality and homoscedasticity were confirmed, the results of each group were analyzed using one-way analysis of variance and then subjected to Dunnett's multiple comparison. When normality but not homoscedasticity was confirmed, the results were subjected to a Welch and Holm's multiple comparison test, and when neither was confirmed, results were analyzed with a Kruskal–Wallis rank sum test and then Holm's multiple comparison test. For determining significance, the rate was set at 5% or lower (*p* < 0.05). For all statistical analyses, IBM SPSS Statistics, version 23, was used.

## 4. Results

### 4.1. Physical Characteristics of Subjects before Starting Daily Consumption and Measurements of Investigated Factors

The physical characteristics of the subjects before starting consumption of the test beverages are shown in Table 2 and values for the investigated factors are presented in Table 3. In the LECA group, there were 2 (7.1%) with less than 5 years after menopause and 19 (67.9%) with 5 or more years after menopause, while 7 (25.0%) were unknown, while those in the LE group were 1 (3.6%), 25 (89.3%), and 2 (8.3%), respectively, and in the control group were 2 (8.7%), 19 (82.6%), and 2 (8.7%), respectively. There were no significant differences among the groups at the baseline.

### 4.2. Changes in BMD

**Figure 2:**
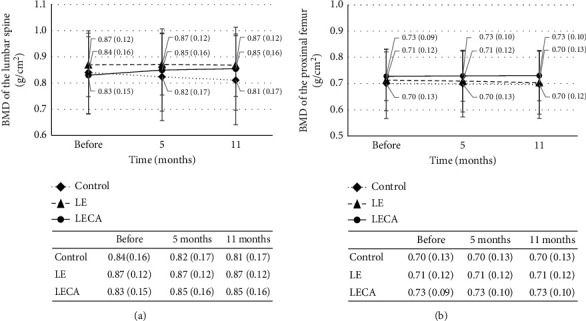
Relationship between ingested product and BMD^1^. (a) BMD of lumbar spine. (b) BMD of proximal femur. Values are shown as the mean (SD) with error bars. Dotted line is the control group, dashed line is the LE group, and solid line is the LECA group. Two-way factorial analysis of variance was performed with type of ingested product and intervention period as factors. *p* values are shown for ingested product (drink), intervention period (time), and interaction. (a) Drink: *p* = 0.666; time: *p* = 0.956; interaction: *p* < 0.001. (b) Drink: *p* = 0.771; time: *p* = 0.413; interaction: *p* < 0.0011. BMD: bone mineral density. SD: standard deviation. The LE group received a nonsupplemented lemon beverage containing citric acid. The LECA group received a lemon beverage containing citric acid supplemented with Ca.

There were no significant differences for mean BMD of the lumbar spine among the three subject groups (*p* = 0.666) or time points (*p* = 0.956). However, there was a significant intervention effect noted (*p* < 0.001) (Figure 2(a)). When the ratio of change from the baseline was calculated for lumbar spine BMD after 11 months of intervention, a positive value was obtained for the LECA group (0.03), while negative values were obtained for the LE and control groups (LE: −0.00; control: −0.04). The differences for these values between the LECA and control, between the LE and control, and between the LECA and LE groups were all significant (*p* < 0.001) (Table 4).

Similarly, there were no significant differences for mean BMD of the proximal femur among the three subject groups (*p* = 0.771) or among different time points (*p* = 0.413). Again, the effects of intervention were significant (*p* < 0.001) (Figure 2(b)). The ratio of change from the baseline calculated for proximal femur BMD after 11 months of ingestion was positive for the LECA group (0.00) but negative for the LE and control groups (LE: −0.02; control: −0.02). The differences between the LECA and control groups and between the LECA and LE groups for this ratio were significant (*p* < 0.001; *p* = 0.001) (Table 4).

### 4.3. Changes in Concentrations of Bone Metabolism Markers

**Figure 3:**
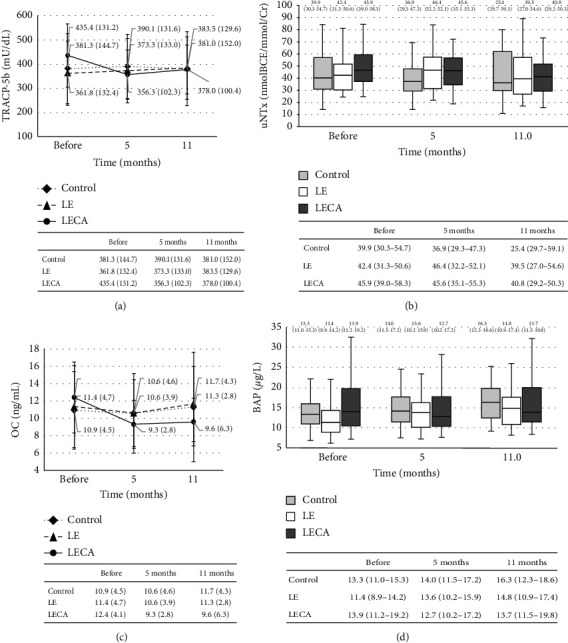
Relationship between ingested product and bone resorption markers. (a) Changes in TRACP-5b concentration. (b) Changes in uNTx for each group (chronological). (c) Changes in OC concentration. (d) Changes in BAP concentration for each group (chronological). (a, c) Values are shown as the mean (SD) with error bars. Dotted line is the control group, dashed line is the LE group, and solid line is the LECA group. Two-way factorial analysis of variance was performed with type of ingested product and intervention period as factors. *p* values for ingested product (drink), intervention period (time), and interaction are shown (^†^). (a) Drink: *p* = 0.840; time: *p* = 0.967; interaction: *p* < 0.001^†^. (c) Drink: *p* = 0.788; time: *p* = 0.254; interaction: *p* = 0.006^†^. (b, d) Values are shown as the median (interquartile range) (25%–75%) with error bars. Light grey box is the control group, blank box is the LE group, and dark grey box is the LECA group. Friedman's test with type of ingested product and intervention period as factors was used. *p* values for each group are shown (^‡^). (b) Control: *p* = 0.401^‡^; LE: *p* = 0.248^‡^; LECA: *p* = 0.131^‡^. (d) Control: *p* < 0.001^‡^; LE: *p* < 0.001^‡^; LECA: *p* < 0.001^‡^. TRACP-5b: tartrate-resistant acid phosphatase type 5b; uNTx: urinary type I collagen cross-linked N-telopeptide; OC: osteocalcin; BAP: bone alkaline phosphatase.

There was no statistically significant difference for mean TRACP-5b concentration observed among the three groups or for the intervention period (*p* = 0.840 and *p* = 0.967, respectively). However, the effects of intervention were significant (*p* < 0.001) (Figure 3(a)). When the ratio of change from baseline was calculated for TRACP-5b level after 11 months of intervention, a negative value was obtained for the LECA group (−0.10), whereas positive values were obtained for the LE and control groups (LE: 0.08; control: 0.03). The difference in these values between the LECA and control groups and between the LECA and LE groups was significant (*p* = 0.002; *p* = 0.003) (Table 4). The concentration of u-NTx was not significantly different during the study period in all three subject groups (Figure 3(b)). Similarly, there were no significant differences among them for ratio of change from baseline after the 11-month intervention (Table 4).

As for the mean concentration of OC, there was no significant difference among the three subject groups (*p* = 0.788) or different time points (*p* = 0.254), while the effects of intervention were significant (*p* = 0.006) (Figure 3(c)). When the ratio of change from the baseline was determined for OC level after 11 months of intervention, the value obtained for the LECA group (−0.27) was significantly (*p* < 0.001) lower than that for the LE group (−0.02) (Table 4). In the case of BAP concentration, a significant (*p* < 0.001) difference during the study period was noted in all three subject groups (Figure 3(d)). The ratio of change from baseline calculated for BAP concentration after 11 months of intervention was positive in all three groups (LECA: 0.03, LE: 0.26, and control: 0.17). However, the LECA group showed a significantly lower value than both the control (*p* = 0.007) and LE (*p* < 0.001) groups (Table 4).

**Figure 4:**
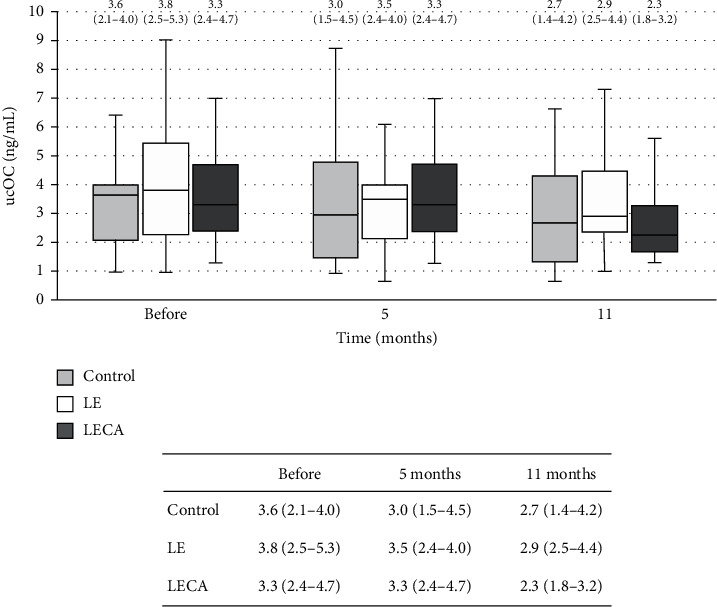
Changes in ucOC concentrations in each group (chronological). Values are shown as the median (interquartile range) (25%–75%) with error bars. Light grey box is the control group, blank box is the LE group, and dark grey box is the LECA group. Friedman's test with type of ingested product and intervention period as factors was used. *p* values for each group are shown (^‡^). Control: *p* = 0.664^‡^; LE: *p* = 0.214^‡^; LECA: *p* < 0.001^‡^. ucOC: undercarboxylated osteocalcin.

Finally, ucOC concentration in the LECA group showed significant (*p* < 0.001) differences during the study period (Figure 4). However, there was no significant difference for ratio of change from the baseline after the 11-month intervention (Table 4).

## 5. Changes in Concentrations of HS-PTH and Serum Ca

**Figure 5:**
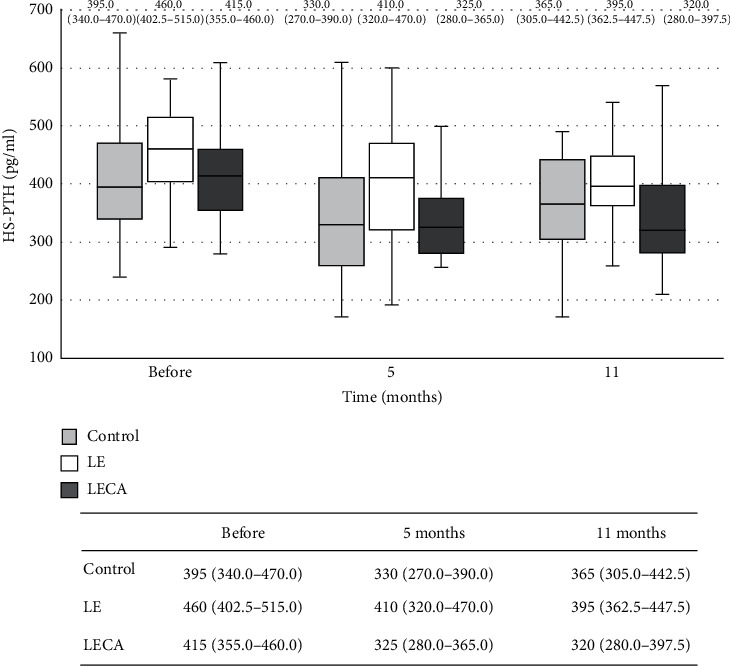
Changes in HS-PTH concentrations in each group (chronological). Values are shown as the median (interquartile range) (25%–75%) with error bars. Light grey box is the control group, blank box is the LE group, and dark grey box is the LECA group. Friedman's test with type of ingested product and intervention period as factors was used. *p* values for each group are shown (^‡^). Control: *p* = 0.003^‡^; LE: *p* < 0.001^‡^; LECA: *p* < 0.001^‡^. HS-PTH: highly sensitive parathyroid hormone.

**Figure 6:**
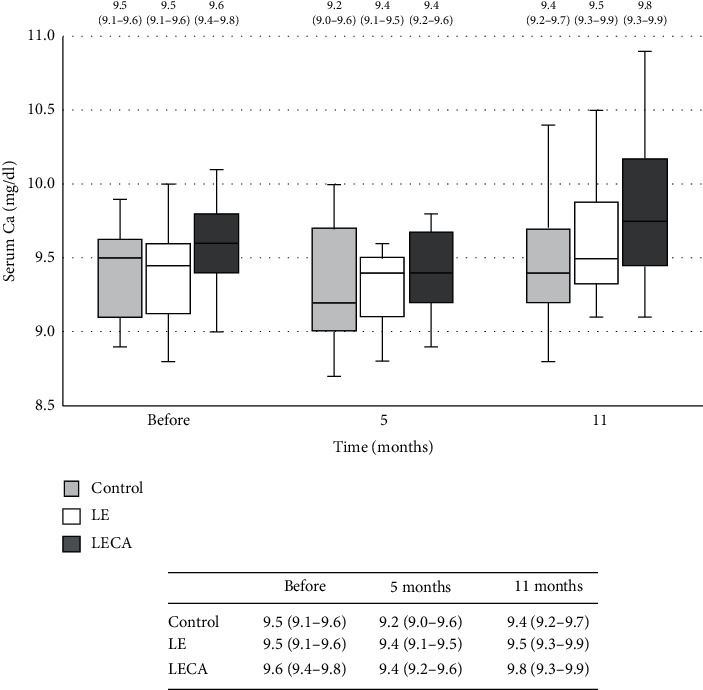
Changes in serum total Ca concentration in each group (chronological). Values are shown as the median (interquartile range) (25%–75%) with error bars. Light grey box is the control group, blank box is the LE group, and dark grey box is the LECA group. Friedman's test with type of ingested product and intervention period as factors was used. *p* values for each group are shown (^‡^). Control: *p* = 0.616^‡^; LE: *p* < 0.001^‡^; LECA: *p* < 0.001^‡^. Ca: calcium.

There was a statistically significant difference for mean HS-PTH concentration among the three groups as well as the intervention period (LECA: <0.001, LE: <0.001, and control: 0.003) (Figure 5). The ratio of change from baseline calculated for HS-PTH after 11 months of intervention was negative for all three groups (LECA: −0.19, LE: −0.11, control: −0.08) and though the LECA group showed a significantly lower value than the control group (*p* = 0.004) (Table 4). As for serum Ca concentration, the LECA and LE groups showed significant (*p* < 0.001) differences during the study period. The ratio of change from the baseline for serum Ca concentration after 11 months of intervention was positive in all three groups (LECA: 0.03, LE: 0.03, and control: 0.01) (Figure 6). However, there were no significant differences among the groups for the ratio of change in serum Ca level from the baseline to the end of the 11-month intervention (Table 4).

## 6. Discussion

For prevention of postmenopausal osteoporosis [19–21], it is important to suppress increased bone metabolic turnover induced by reduced estrogen for maintaining BMD. In the present study, the effects of the long-term intervention (11 months) with a Ca-supplemented as well as nonsupplemented lemon beverage containing citric acid were examined. The results of comprehensive evaluations of bone (lumbar spine, proximal femur) using BMD and biomarkers of bone turnover in healthy postmenopausal women (mean age 64.7 ± 6.0 years) revealed suppression of increased bone metabolic turnover and BMD maintenance in the LECA group.

We considered that the chelating action of citric acid, the major component causing sourness in lemons, might have been involved in the effects seen in this study. Lemon juice is known to contain abundant citric acid (1.44 g/oz) [22], and its chelation is well known to enhance resorption of minerals such as Ca and iron by making them more water-soluble [23–25]. Enhanced resorption of Ca is thought to lead to suppression of increases in bone resorption and bone formation marker levels, resulting in suppression of increased bone metabolic turnover, as seen in the LECA group.

A previous study reported that bone formation markers were reduced following a decrease in bone resorption markers, because the reduction in the former was found to be in response to suppression of bone resorption by a coupling phenomenon [18]. In the present LECA group, the concentration of TRACP-5b dropped to a negative value as compared to the baseline after 11 months of consumption of the test beverage, whereas the BAP concentration was scarcely increased. These results suggest that suppression of increased bone formation might be delayed more than that of bone resorption. In a previous intervention study that utilized an antiosteoporotic agent [26], no significant changes were detected in ΣGS/D after 6 months of treatment. In contrast, similar treatment approaches have resulted in favorable clinical effects, such as decreased loss of BMD or increase in BMD after 1 to 2 years of intervention [14, 15, 27, 28]. Therefore, for validating the osteoporosis prevention effect, it is considered that a long-term intervention period of at least 1 year might be required. Nevertheless, the present results indicate that the changes in both bone resorption and bone formation markers detected in our subjects might be significant for understanding bone condition.

In studies performed by Smith et al. [29] and Reid et al. [30], Ca supplementation (1000–1500 mg/day) in postmenopausal women lowered the level of BMD loss. Although the amount of Ca contained in the Ca-supplemented lemon beverages used in the present study was much lower at 350 mg/bottle as compared to those studies [30, 31], the interaction effect was significant on mean BMD of the lumbar spine and proximal femur. Calculation of the ratio of change from the baseline showed that the differences in those values for lumbar spine BMD between the LECA and control and LE groups were significant after 11 months of intervention. Moreover, there were also significant differences between the LECA and control and LE groups for proximal femur BMD. This may have been due to the following reasons. When the ratios of change from baseline were calculated for TRACP-5b levels after 11 months of intervention, negative and positive values were obtained for the LECA and control groups, respectively, and the difference between them was significant. As for BAP concentration, the ratio of change from the baseline after 11 months of intervention was positive for all three subject groups. However, the LECA group showed a significantly lower value than both the control (*p* = 0.007) and LE (*p* < 0.001) groups, which might have been the result of attenuation of high-turnover bone metabolism. Accordingly, the BMD value in the LECA group was thought to be maintained despite the time of decline.

Generally, serum Ca level in postmenopausal women is easily reduced by decreased Ca resorption from the gastrointestinal tract due to aging as well as from impaired activity of vitamin D in the kidney induced by a low level associated with decreased intake or reduced in vivo production from lack of UV exposure [32]. It is suggested that the effects noted in the present LECA group were associated with maintenance of serum Ca at a consistent level, as Ca resorption was accelerated by the chelation response of citric acid during the period of consumption. Consequently, since serum Ca level was maintained, its supply from bone was not necessary, resulting in suppression of osteoclastic bone resorption in that group. In association with this finding, it is considered that the synthetic function of osteoblasts might be suppressed, leading to suppression of bone formation. Thus, high-turnover-type bone metabolism induced by menopause [23, 33] might have been suppressed in the LECA group subjects.

A bone fracture caused by osteoporosis frequently occurs in the vertebral body, proximal femur, lower leg, and distal end of the radius, of which a vertebral body fracture occurs most frequently [18], while a proximal femur fracture significantly increases the risk of reduced activities of daily living and bedridden status [34]. Those bones are composed of cortical and cancellous bone tissue, whereas the lumbar spine is composed mainly of cancellous bone and the femur mainly of cortical bone [35]. Therefore, when evaluating bone mineral density, evaluation of the lumbar spine and femur is recommended. In the present study, to validate the effects of simultaneous administration of citric acid and Ca, evaluation of BMD was performed in two sites, the lumbar spine (L2-L4) and proximal femur. The ratio of change from the baseline in both was significantly higher in the LECA group than the control group, indicating that administration of a Ca-supplemented lemon beverage is effective to prevent a reduction in BMD in both types of bone tissue.

Additionally, we found that the concentration of the bone matrix-related marker ucOC was significantly reduced in the LECA group after 11 months of intervention. ucOC, which is produced in the absence of vitamin K in blood and represents the uncarboxylated form, is unable to bind Ca [36]; thus, a high ucOC level shows the low vitamin K status of the examined bone, that is, a condition in which bone formation is inhibited and bone resorption is promoted [23, 37]. It has been reported that the concentration of ucOC was increased with older age in women [38]. Under a condition of high bone metabolic turnover with increased bone formation and resorption, it is speculated that vitamin K does not act on bone and ucOC is released into the bloodstream without being incorporated into the bone matrix, resulting in an increased concentration of ucOC in serum. That suggests that vitamin K in bone tissue is involved in both inhibition of bone resorption and promotion of bone formation. We found increases in bone resorption and suppression of bone formation markers in the LECA group after 11 months of test beverage ingestion. Although clarification of the relationship of ucOC concentration to bone metabolic turnover was not the purpose of this study, it can be speculated that vitamin K may have functions related to bone resorption suppression and bone formation facilitation, which might have been related to our finding that bone mineral density was maintained in the LECA group despite the period of decrease.

Parathyroid hormone and calcitonin are factors directly involved in bone metabolism. Calcitonin and estrogen suppress bone mineral elution to protect bone, whereas parathyroid hormone acts to facilitate that elution [8]. Under a normal condition, these hormones maintain a balance with each other to regulate bone metabolism. However, with aging, along with the decreased secretion of calcitonin and estrogen, and an increase in that of parathyroid hormone, the development of osteoporosis is considered to be activated. In the present subjects, the mean concentrations of highly sensitive PTH and serum Ca were within normal ranges; thus, hyperparathyroidism and hypercalcemia were considered to be absent. Nevertheless, there is potential for bias, as our subjects were considered to be in a healthy condition or highly health-conscious, as they were not recruited from patients receiving treatments at a medical institution or hospitalized, but rather individuals who volunteered to participate in a health investigation.

The test beverages for both the LECA and LE groups contained 30 ml of lemon juice, while the Ca-supplemented lemon beverage given to the LECA group also contained 350 mg of Ca. Domoto et al. [39] reported that daily consumption of 30 mL of lemon juice had an effect to significantly decrease blood pressure. As for the use of fruit juice in such a study, safety problems are unlikely as there have been no specific reports of potential health hazards with normal ingestion of usual types of food. The standard Ca intake per day for adult women is about 700 mg [29]. Taking into account Ca intake from daily meals, we set the amount in the beverage at 350 mg, half of the standard intake. However, since the individual diet habits of our subjects were not recorded, possible heterogeneity because of lifestyle habits is undeniable and should be an issue for analysis in a future investigation. The physical conditions of the subjects in the present study were approximative to the mean of the same age group reported in another investigation [40]. Accordingly, at least in view of the physical condition of the cohort, the present study is thought to provide valid information for postmenopausal women in general.

The present study protocol is considered to be unique. It was conducted as an 11-month interventional trial using lemon beverages containing citric acid with or without Ca supplementation to comprehensively evaluate trunk bones (lumbar spine; proximal femur) for BMD maintenance, suppression of high bone metabolic turnover, and the activities of vitamin K on bone resorption suppression and bone formation facilitation. However, there was no group that received Ca alone, and measurements of intracellular Ca concentration and Ca in excrement would provide important data related to the mechanism by which citric acid increases Ca absorption. That is a limit of this study. The HS-PTH and serum total Ca concentrations in the present cohort were within normal ranges. In the control and LE groups, serum Ca concentration was maintained even though bone resorption remained high, suggesting that Ca was excreted from bone. Since the serum Ca concentration was maintained in the LECA group despite suppression of bone resorption, Ca absorption by citric acid may have been promoted, though that is only speculation. The effects on bone were inferred from changes in bone resorption markers [41], as a constant serum Ca level is known to be maintained throughout the process of bone remodeling.

In conclusion, for investigating the effects of osteoporosis prevention, a long-term intervention protocol was performed for 11 months with postmenopausal women. The results showed that Ca supplementation in a lemon beverage containing citric acid suppressed bone resorption by the chelating action of citric acid along with acceleration of Ca resorption. Furthermore, bone formation caused by osteoclastic response was suppressed along with suppression of bone resorption. Accordingly, we speculated that high-turnover-type bone metabolism might have been inhibited. These novel findings are considered to reflect BMD in both the lumbar spine and proximal femur. Consumption of a Ca-supplemented lemon beverage containing citric acid might be useful for prevention of osteoporosis in postmenopausal women.

## 7. Conclusion

In this study of osteoporosis prevention, postmenopausal women underwent a long-term intervention for 11 months. It was shown that Ca supplementation in a lemon beverage containing citric acid suppressed bone resorption by the chelating action of citric acid along with acceleration of Ca resorption. Additionally, suppression of bone formation caused by osteoclastic response was noted along with suppressed bone resorption. We speculated that high-turnover-type bone metabolism might have been inhibited in the subjects. These novel findings are considered to reflect BMD in both the lumbar spine and proximal femur. For prevention of osteoporosis in postmenopausal women, development of a Ca-supplemented lemon beverage containing citric acid might be promising.

## Figures and Tables

**Table 1:** Test beverage compositions.

		LE group	LECA group
Energy	(kcal/100 mL)	7	17
Protein	(g/100 mL)	0	0
Fat	(g/100 mL)	0	0
Carbohydrate	(g/100 mL)	1.8	4.4
Ash	(g/100 mL)	0	0.3
Sodium	(mg/100 mL)	0	1.4
Lemon juice	(mL/100 mL)	10.4	10.4
Calcium\	(mg/100 mL)	0	120.7

The LE group received a nonsupplemented lemon beverage containing citric acid (∼1.5 g/bottle). The LECA group received a lemon beverage containing citric acid (∼1.5 g/bottle) supplemented with Ca.

**Table 2:** Subject physical characteristics.

	Subject groups			Total (*n* = 79)	*p* value
	Control (*n* = 23)	LE (*n* = 28)	LECA (*n* = 28)		
Age, years	63.9 (7.1)	64.9 (5.8)	65.2 (5.2)	64.7 (6.0)	0.676
Years after menopause	13.0 (6.9)	15.1 (9.2)	16.4 (7.6)	14.9 (8.1)	0.258
Height, cm	154.3 (5.4)	154.5 (4.6)	152.8 (5.6)	153.9 (5.2)	0.559
Baseline weight, kg	50.8 (10.1)	53.4 (8.5)	52.3 (7.2)	52.3 (8.5)	0.255
Baseline BMI, kg/m^2^	21.6 (3.5)	22.2 (3.5)	22.4 (2.8)	22.1 (3.2)	0.534

Values are shown as the mean (SD). *p* value is a result obtained by one-way analysis of variance. There were no significant differences for any of these variables among the three subject groups. The LE group received a nonsupplemented lemon beverage containing citric acid. The LECA group received a lemon beverage containing citric acid supplemented with Ca. BMI: body mass index. SD: standard deviation.

**Table 3:** Measurements before starting intervention.

		Subject groups			Total (*n* = 79)	*p* value
		Control (*n* = 23)	LE (*n* = 28)	LECA (*n* = 28)		
Bone mineral density	Lumbar spine (g/cm^2^)	0.84 (0.16)	0.87 (0.12)	0.83 (015)	0.85 (0.14)	0.567^a^
	T-score (SD)	−1.43 (0.25)	−1.13 (0.22)	−1.45 (0.22)	−1.33 (1.17)	0.534^a^
	Proximal femur (g/cm^2^)	0.70 (0.13)	0.71 (0.12)	0.73 (0.09)	0.72 (0.11)	0.738^a^
	T-score (SD)	−1.45 (0.22)	−.136 (0.19)	−1.25 (0.19)	−1.35 (1.03)	0.793^a^

Bone metabolism marker	TRACP-5b (mU/dL)	397.8 (144.7)	361.8 (132.4)	435.4 (131.2)	398.4 (137.5)	0.134^a^
	uNTx ^*∗*^ (nmol BCE/mmol/Cr)	39.9 (30.3–56.5)	42.5 (25.7–51.4)	45.9 (37.5–59.1)	43.3 (32.0–55.0)	0.492^b^
	BAP ^*∗*^ (*μ*g/L)	13.3 (10.9–15.9)	11.4 (8.9–14.3)	13.9 (10.4–19.7)	12.5 (9.7–16.2)	0.057^b^
	OC (ng/mL)	11.4 (4.6)	11.4 (4.7)	12.4 (4.1)	11.7 (4.4)	0.613^a^

Bone matrix-related marker	ucOC ^*∗*^ (ng/mL)	3.6 (2.1–4.0)	3.8 (2.3–5.4)	3.3 (2.4–4.7)	3.6 (2.2–4.8)	0.674^b^

HS-PTH ^*∗*^ (pg/mL)		395.0 (340.0–470.0)	460.0 (402.5–515.0)	415.0 (355.0–460.0)	420.0 (370.0–482.5)	0.111^b^

Serum total Ca ^*∗*^ (mg/dL)		9.5 (9.1–9.6)	9.5 (9.1–9.6)	9.6 (9.4–9.8)	9.5 (9.3–9.7)	0.069^b^

Values are shown as the mean (SD).  ^*∗*^Median (25%–75%) because normality was not shown. ^a^Result obtained by one-way analysis of variance. ^b^Result obtained by Kruskal–Wallis test. TRACP-5b: tartrate-resistant acid phosphatase type 5b; uNTx: urinary type I collagen cross-linked N-telopeptide; BAP: bone alkaline phosphatase; OC: osteocalcin; ucOC: undercarboxylated osteocalcin; HS-PTH: highly sensitive parathyroid hormone; Ca: calcium.

**Table 4:** Ratio of change after 11 months of intervention.

Ratio of change		Subject group			*p* value			*p* value
		Control (*n* = 23)	LE (*n* = 28)	LECA (*n* = 28)	Versus LE	Versus LECA	LE versus LECA	
Bone mineral density	Lumbar spine (g/cm^2^)	−0.04 (0.03)	−0.00 (0.02)	0.03 (0.03)	<0.001^a^	<0.001^a^	<0.001^a^	<0.001^a^
	Proximal femur (g/cm^2^)	−0.02 (0.02)	−0.02 (0.02)	0.00 (0.01)	0.995^a^	<0.001^a^	0.001^a^	<0.001^a^

Bone metabolism marker	TRACP-5b (mU/dL)	0.03 (0.27)	0.08 (0.17)	−0.10 (0.16)	0.685^a^	0.002^a^	0.003^a^	0.004^a^
	u-NTx (nmol BCE/mmol/Cr) ^*∗*^	0.00 (−0.45–0.30)	−0.01 (−0.29–0.22)	−0.16 (−0.38–0.03)	1.000^c^	1.000^c^	1.000^c^	0.573^b^
	BAP (*μ*g/L)	0.17 (0.02)	0.26 (0.17)	0.03 (0.15)	0.135^a^	0.007^a^	<0.001^a^	<0.001^a^
	OC (ng/ml) ^*∗*^	0.00 (−0.29–0.21)	−0.02 (−0.12–0.15)	−0.27 (−0.36–0.10)	0.590^c^	0.060^c^	<0.001^c^	0.001^b^

Bone matrix-related marker	ucOC (ng/mL) ^*∗*^	−0.10 (−0.33–0.12)	−0.14 (−0.25–0.12)	−0.22 (−0.43–0.03)	0.580^c^	0.530^c^	0.150^c^	0.152^b^

HS-PTH (pg/ml)		−0.08 (0.21)	−0.11 (0.15)	−0.19 (0.16)	0.648^a^	0.040^a^	0.164^a^	0.056^a^

Serum total Ca (mg/dl) ^*∗*^		0.01 (−0.02–0.02)	0.03 (0.00–0.04)	0.03 (−0.01–0.04)	0.367^c^	0.349^c^	0.999^c^	0.102^b^

Values are shown as the mean (SD).  ^*∗*^Median (25%–75%), because normality was not shown. ^a^Result obtained by Dunnett's multiple comparison test after one-way analysis of variance. ^b^Result obtained by Kruskal–Wallis test. ^c^Result obtained by the *p* value adjustment method (Holm's) after the Kruskal–Wallis test.

## Data Availability

The present data cannot be shared publicly because the datasets have ethical or legal restrictions for public deposition owing to inclusion of sensitive information from the human participants. Based on ethical guidelines in Japan, the ethical review board of the Faculty of Health and Welfare, Prefectural University of Hiroshima, imposed restrictions on the data collected in this study. The obtained data and materials were used only for the present study and are available only to the researchers who participated in the study project.

## Supplementary Materials

Supplementary Materials The document “CONSORT checklist (ikeda).docx” was used to confirm this study.

## References

[1] Ohta H., Nozawa S. (1995). Postmenopausal osteoporosis. -Mainly on a drop of estrogen and sthenia of the bone resorption. *Journal of Clinical and Experimental Medicine*.

[2] Kanis J. A., Johnell O., Oden A. (2000). Long-term risk of osteoporotic fracture in malmö. *Osteoporosis International*.

[3] Howe T. E., Shea B., Dawson L. J. (2011). Exercise for preventing and treating osteoporosis in postmenopausal women. *Cochrane Database System Review*.

[4] Nguyen T. V., Center J. R., Eisman J. A. (2004). Osteoporosis: underrated, underdiagnosed and undertreated. *Medical Journal of Australia*.

[5] Hirota T., Nara M., Ohguri M., Manago E., Hirota K. (1992). Effect of diet and lifestyle on bone mass in Asian young women. *The American Journal of Clinical Nutrition*.

[6] Jehle S., Hulter H. N., Krapf R. (2013). Effect of potassium citrate on bone density, microarchitecture, and fracture risk in healthy older adults without osteoporosis: a randomized controlled trial. *The Journal of Clinical Endocrinology & Metabolism*.

[7] Hua P., Xiong Y., Yu Z., Liu B., Zhao L. (2019). Effect of chlorella pyrenoidosa protein hydrolysate-calcium chelate on calcium absorption metabolism and gut microbiota composition in low-calcium diet-fed rats. *Marine Drugs*.

[8] Nii Y., Fukuta K., Kiyokage R., Sakai K., Yamamoto S. (1997). In vitro effects of citrus fruit juices on solubilization of calcium from shirasuboshi(boiled and semi-dried whitebaits). *Nippon Eiyo Shokuryo Gakkaishi*.

[9] Ikeda H., Iida T., Hiramitsu M. (2018). Effects of lemon beverages on bone metabolism and bone mineral density in postmenopausal women: a double-blind, controlled intervention study with Ca-supplemented and unsupplemented lemon beverages. *Open Journal of Preventive Medicine*.

[10] Parfitt A. M. (1980). Morphologic basis of bone mineral measurement: ransient and steady state effects of treatment in osteoporosis. *Miner Electrolyte Metab*.

[11] Schott A. M., Cormier C., Hans D. (1998). How hip and whole-body mineral density predict hip fracture in elderly women: the EPIDOS Prospective Study. *Osteoporos Lnt*.

[12] Lewiecki E. M., Watts N. B., McClung M. R. (2004). Official positions of the international society for clinical densitometry. *The Journal of Clinical Endocrinology & Metabolism*.

[13] Cummings S. R., Browner W., Cummings S. R. (1993). Bone density at various sites for prediction of hip fractures. *The Lancet*.

[14] Purwosunu Y., Rachman I. A., Reksoprodjo S., Sekizawa A. (2006). Vitamin K2 treatment for postmenopausal osteoporosis in Indonesia. *Journal of Obstetrics and Gynaecology Research*.

[15] Jiang Y., Lin Z., Zhang, Lan Z., Zhang (2014). Menatetrenone versus alfacalcidol in the treatment of Chinese postmenopausal women with osteoporosis: a multicenter, randomized, double- blinded, double- dummy, positive drug-controlled clinical trial. *Clinical Interventions in Aging*.

[16] Bolland M. J., Leung W., Tai V. (2015). Calcium intake and risk of fracture: systematic review. *British Medical Journal*.

[17] Rebecca D. J., LaCroix A. Z., Gass M. (2006). Calcium plus vitamin D supplementation and the risk of fractures. *The New England Journal of Medicine*.

[18] Japan Osteoporosis Foundation (2015). *Making Committee of the Prevention and Treatment Guidelines on Osteoporosis. The Prevention and Treatment Guidelines on Osteoporosis 2015 Version*.

[19] Albright F., Smith P. H., Richardson A. M. (1941). Postmenopausal osteoporosis. *Journal of the American Medical Association*.

[20] Gorai I. (1997). Change of bone metabolism with ageing –in relation to involvement of estrogen withdrawal. *Acta Obstetrica et Gynaecologica Japonica*.

[21] Iida T., Ishizaki F., Hayashi M. (2001). Effect of menopause on bone metabolism indices and bone mineral density. -study of the usefulness of bone metabolism indices. *Japanese Journal of Clinical Chemistry*.

[22] Penniston K. L., Nakada S. Y., Holmes R. P., Assimos D. G. (2008). Quantitative assessment of citric acid in lemon juice, lime juice, and commercially-available fruit juice products. *Journal of Endourology*.

[23] Lacour B., Taedivel S., Drüeke T. (1997). Stimulation by citric acid of calcium and phosphorous bioavailability in rats fed a calcium-rich diet. *Miner Electrolyte Metab*.

[24] Pak C. Y. C., Harvey J. A., HSU M. C. (1987). Enhanced calcium bioavailability from a solubilized form of calcium citrate. *The Journal of Clinical Endocrinology & Metabolism*.

[25] Miller J. Z., Smith D. L., Flora L., Slemenda C., Jiang X. Y., Johnston C. C. (1988). Calcium absorption from calcium carbonate and a new form of calcium (CCM) in healthy male and female adolescents. *The American Journal of Clinical Nutrition*.

[26] Orimo H., Shiraki M., Tomita A., Morii H., Fujita T., Ohata M. (1998). Effects of menatetrenone on the bone and calcium metabolism in osteoporosis: a double-blind placebo-controlled study. *Journal of Bone and Mineral Metabolism*.

[27] Shiraki M., Shiraki Y., Aoki C., Miura M. (2000). Vitamin K2 (menatetrenone) effectively prevents fractures and sustains lumbar bone mineral density in osteoporosis. *Journal of Bone and Mineral Research: The Official Journal of the American Society for Bone and Mineral Research*.

[28] Ushiroyama T., Ikeda A., Ueki M. (2002). Effect of continuous combined therapy with vitamin K2 and vitamin D3 on bone mineral density and coagulofibrinolysis function in postmenopausal women. *Maturitas*.

[29] Smith E. L., Gilligan C., Smith P. E., Sempos C. T. (1989). Calcium supplementation and bone loss in middle-aged women. *The American Journal of Clinical Nutrition*.

[30] Reid I. R., Ames R. W., Evans M. C., Gamble G. D., Sharpe S. J. (1995). Long-term effects of calcium supplementation on bone loss and fractures in postmenopausal women: a randomized controlled trial. *The American Journal of Medicine*.

[31] Zou Z.-Y., Lin X.-M., Xu X.-R. (2009). Evaluation of milk basic protein supplementation on bone density and bone metabolism in Chinese young women. *European Journal of Nutrition*.

[32] Banba N. (2008). Change by the aging -Internal secretion metabolism-. *Dokkyo Journal of Medical Sciences*.

[33] Riggs B. L. (1987). Pathogenesis of osteoporosis. *American Journal of Obstetrics and Gynecology*.

[34] Yamamoto K. (1996). [The effects of fracture of the femoral neck on the lives of patients and their families]. *Acta Scientiarvm Valettvdinis Universitatis Praefectvralis Ibarakiensis*.

[35] Fukunaga M., Sone T. (2005). [The measurement of the bone density]. *The Journal of the Japanese Society of Internal Medicine*.

[36] Kishikawa Y. (2011). Actual situation of ucOC examination in osteoporosis treatment. *Orthopedics & Traumatology*.

[37] Villa J. K. D., Diaz M. A. N., Pizziolo V. R., Martino H. S. D. (2017). Effect of vitamin K in bone metabolism and vascular calcification: a review of mechanisms of action and evidences. *Critical Reviews in Food Science and Nutrition*.

[38] Liu G., Peacock M. (1998). Age-related changes in serum undercarboxylated osteocalcin and its relationships with bone density, bone quality, and hip fracture. *Calcified Tissue International*.

[39] Domoto T., Ishihara K., Miyake Y. (2010). Effect of daily lemon intake on a range of parameters related to metabolic syndrome. *Health Sciences*.

[40] Ministry of Health, Labour and Welfare (2017). *Public Welfare Statistics Handbook, “Mean of Height, the Weight, Sex, Annual× Age Distinction*.

[41] Peacock M. (2010). Calcium metabolism in health and disease. *Clinical Journal of the American Society of Nephrology*.

