# Supplementary material for: Effects of Lemon Beverage Containing Citric Acid with Calcium Supplementation on Bone Metabolism and Mineral Density in Postmenopausal Women: Double-Blind 11-Month Intervention Study
Source: J Nutr Metab. 2021 Feb 24;2021:8824753. doi: 10.1155/2021/8824753 (PMC7935594; doi:10.1155/2021/8824753)
Supplement: Supplementary Materials — The document “CONSORT checklist (ikeda).docx” was used to confirm this study. [file 8824753.f1.docx]

5Table 1: CONSORT 2010 checklist of information to include when reporting a within-person randomised trial. For within-person trials, a group is the set of participants’ body sites that was allocated a particular intervention.

| **Section/Topic** | **Item no.** | **Standard CONSORT Checklist item** | **Extension for within-person trials** | **Page no.** |
| --- | --- | --- | --- | --- |
| **Title and abstract** | | | |  |
|  | 1a | Identification as a randomised trial in the title | Identification as a within-person randomised trial in the title | 1 (Title) |
|  | 1b | Structured summary of trial design, methods, results, and conclusions (for specific guidance see CONSORT for abstracts[3]) | Specify a within-person design and report all information outlined in table 2 | 1 (Abstract) |
| **Introduction** | | | |  |
| Background and objectives | 2a | Scientific background and explanation of rationale |  | 2 (Introduction) |
|  | 2b | Specific objectives or hypotheses |  | 2 (Introduction, paragraph 4) |
| **Methods** | | | |  |
| Trial design | 3a | Description of trial design (such as parallel, factorial) including allocation ratio | Rationale for using a within-person design and identification of body sites | 2-5 (Methods, subjects) |
|  | 3b | Important changes to methods after trial commencement (such as eligibility criteria), with reasons |  | N/A |
| Participants | 4a | Eligibility criteria for participants | Eligibility criteria for body sites | 3 (Materials and Methods, subjects) |
|  | 4b | Settings and locations where the data were collected |  | 4 (Materials and Methods, Items investigated) |
| Interventions | 5 | The interventions for each group with sufficient details to allow replication, including how and when they were actually administered | Whether interventions were given sequentially or concurrently | 2-3 (Materials and Methods, subjects and Item investigated) |
| Outcomes | 6a | Completely defined pre-specified primary and secondary outcome measures, including how and when they were assessed | Outcomes should be clearly defined as per-site or per-person | 3-7 (Materials and Methods,1. ~5.) |
|  | 6b | Any changes to trial outcomes after the trial commenced, with reasons |  | N/A |
| Sample size | 7a | How sample size was determined | Report the correlation between body sites | 5-6(Materials and Methods, Data analysis) |
|  | 7b | When applicable, explanation of any interim analyses and stopping guidelines |  | N/A |
| Randomisation: | | | |  |
| Sequence generation | 8a | Method used to generate the random allocation sequence |  | 3-5 (Materials and Methods, subjects) |
|  | 8b | Type of randomisation; details of any restriction (such as blocking and block size) | Methods used to determine the allocation sequence of body sites and treatments within an individual (e.g. how first site to be treated was decided) | N/A |
| Allocation concealment mechanism | 9 | Mechanism used to implement the random allocation sequence (such as sequentially numbered containers), describing any steps taken to conceal the sequence until interventions were assigned |  | 3(Materials and Methods, subjects, paragraph 2) |
| Implement-ation | 10 | Who generated the random allocation sequence, who enrolled participants, and who assigned participants to interventions | Replaced by 10a | 4(Materials and Methods, subjects, paragraph 2) |
|  | 10a |  | Who generated the random allocation sequence, who enrolled participants, and who assigned body sites to interventions | 4 (Materials and Methods, subjects, paragraph 2) |
| Blinding (masking) | 11a | If done, who was blinded after assignment to interventions (for example, participants, care providers, those assessing outcomes) and how |  | 4 (Materials and Methods, subjects) |
|  | 11b | If relevant, description of the similarity of interventions |  | N/A |
| Statistical methods | 12a | Statistical methods used to compare groups for primary and secondary outcomes | Statistical methods appropriate for within-person design | 6-7 (Materials and Methods, 5. Data Analysis) |
|  | 12b | Methods for additional analyses, such as subgroup analyses and adjusted analyses |  | N/A |
| **Results** | | | |  |
| Participant flow  (a diagram is strongly recommended) | 13a | For each group, the numbers of participants who were randomly assigned, received intended treatment, and were analysed for the primary outcome | Number of participants and number of body sites at each stage [See Figure 1] | 3-4 (Materials and Methods, Subjects) |
|  | 13b | For each group, losses and exclusions after randomisation, together with reasons | Number of participants and number of body sites lost or excluded at each stage, with reasons | 5-6 (Materials and Methods, 5. Data Analysis)  Figure1: Study flowchart |
| Recruitment | 14a | Dates defining the periods of recruitment and follow-up |  | 3-4 (Materials and Methods, subjects) |
|  | 14b | Why the trial ended or was stopped |  | 3(Materials and Methods, subjects) |
| Baseline data | 15 | A table showing baseline demographic and clinical characteristics for each group | Baseline characteristics for site and individual participants as applicable | 7-8 (Results, 1) |
| Numbers analysed | 16 | For each group, number of participants (denominator) included in each analysis and whether the analysis was by original assigned groups | Number of randomised body sites in each group included in each analysis | 3 (Materials and Methods, subjects) |
| Outcomes and estimation | 17a | For each primary and secondary outcome, results for each group, and the estimated effect size and its precision (such as 95% confidence interval) | Observed correlation between body sites for continuous outcomes and tabulation of paired results for binary outcomes | 8-13 (Results, 2. -3.) |
|  | 17b | For binary outcomes, presentation of both absolute and relative effect sizes is recommended |  | N/A |
| Ancillary analyses | 18 | Results of any other analyses performed, including subgroup analyses and adjusted analyses, distinguishing pre-specified from exploratory |  | 5-6 (Methods, Data Analysis) |
| Harms | 19 | All important harms or unintended effects in each group (for specific guidance see CONSORT for harms) | Harms or unintended effects reported by participant and by body site | N/A |
| **Discussion** | | | |  |
| Limitations | 20 | Trial limitations, addressing sources of potential bias, imprecision, and, if relevant, multiplicity of analyses |  | 16 (Discussion, line465-469) |
| Generalisability | 21 | Generalisability (external validity, applicability) of the trial findings |  | 14 (Discussion, line469-471) |
| Interpretation | 22 | Interpretation consistent with results, balancing benefits and harms, and considering other relevant evidence |  | 13-16 (Discussion) |
| **Other information** | | | |  |
| Registration | 23 | Registration number and name of trial registry |  | 3 (line112) |
| Protocol | 24 | Where the full trial protocol can be accessed, if available |  | N/A |
| Funding | 25 | Sources of funding and other support (such as supply of drugs), role of funders |  | 17 (Funding Statement) |
